# Heavy oil oxidation in the nano-porous medium of synthetic opal

**DOI:** 10.1039/c8ra02822b

**Published:** 2018-05-17

**Authors:** Andrey Galukhin, Dmitrii Bolmatenkov, Yuri Osin

**Affiliations:** Kazan Federal University Russia and_galuhin@mail.ru

## Abstract

Increasing interest to study hydrocarbon behavior in fine porous media, awakened by the shale revolution, requires the application of suitable model porous media. In the current study we prepared nano-porous synthetic opal, profoundly investigated its morphological and textural properties, and studied the kinetics of combustion of heavy oil impregnated into nanopores. Comparison of kinetic parameters of the oil oxidation process for nano-porous and coarse-porous media revealed that nanoconfinement affects the reactivity of oil.

## Abstract 2

In the current study we synthesized nano-porous opal, investigated its morphological and textural properties, and showed that nanoconfinement affects reactivity of oil.

## Graphical abstract

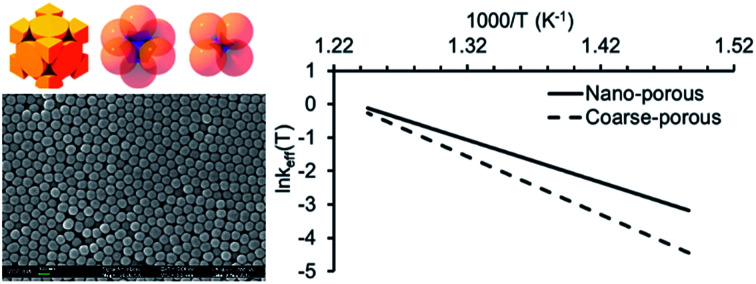

## 1. Introduction

The shale revolution in the petroleum industry started in the USA and resulted in enhanced production of gas and oil from shale deposits. This awakened increasing interest to study gas and fluid behavior in fine porous media. At the moment the influence of micro- and nanoconfinement on the physical properties of hydrocarbons are well presented in the scientific literature. The influence of confinement on crystallization and melting,^1,2^ and on bubble point temperatures^3^ of individual hydrocarbons and their binary mixtures^4–6^ is a well-known phenomenon, as is the influence of porous media properties on the formation of gas hydrates.^7–9^ The general conclusion from these studies is that confinement influences both the thermodynamics and kinetics of physical processes. Unlike physical processes, the influence of confinement on chemical reactions is a rare subject of research.

Air injection is considered as a promising method for enhanced oil recovery for oil production from shale reservoirs (especially from those with high temperature and pressure).^10,11^ Injected air oxidizes part of the hydrocarbons and generates heat and pressure to enhance oil recovery. Thus, heat and mass transfer along with the thermodynamics and kinetics of the chemical reactions are the key elements needed for successful description of the whole process. Currently, the fundamentals of heat^12,13^ and mass^14–16^ transfer in nanoporous media have been studied in greater detail than the influence of nanoconfinement on chemical reactivity, especially for reactions proceeding in the condensed state. Thus, investigation of this will complement existing studies and allow us to raise our understanding of the processes taking place.

Our current study is an attempt to fill this gap: we synthesized a new type of model porous medium and investigated the influence of nanoconfinement on reactivity of heavy oil in the combustion process.

## 2. Materials and methods

### 2.1 Materials

Ammonium hydroxide solution (25% of NH_3_), trichloromethane (99.5%), tetraethylorthosilicate (TEOS, 99.9%, Sigma Aldrich), and ethanol (96%) were used without additional purification. Deionized water (18.2 MΩ) was obtained by Barnstead EASYpure II equipment (Thermo Scientific).

The crude oil used in this research was extracted from an oil core taken from the Ashal’cha oilfield (Volga-Ural basin, Republic of Tatarstan, Russia).^17–19^ The physical properties and elemental and SARA (saturated and aromatic hydrocarbons, resins and asphaltenes) analysis data of crude oil are given in Table 1.

**Table 1:** Physical properties of Ashal’cha heavy oil at 20 °C

Viscosity, mPa s	Density, g cm^−3^	API gravity	Elemental content, %				SARA analysis, %			
11 811	0.97	13.8	C	H	N	S	Saturated	Aromatic	Resins	Asphaltenes
			82.09	10.12	0.63	2.65	26.2 ± 0.5	44.1 ± 0.6	26.3 ± 0.5	4.5 ± 0.3

### 2.2 Preparation of 93 nm-diameter silica spheres

Silica nanoparticles were prepared by a modified Stöber method based on the controllable growth of silica nanoparticles from seeds, as described by Giesche.^20^ Silica nanoparticles were isolated by centrifugation (10 000 rpm) and were washed consistently with ethanol, water–ethanol solutions (water to ethanol ratios were 1 : 3, 1 : 1 and 3 : 1) and deionized water. The dried silica spheres were then calcined in a furnace at 600 °C for 12 hours, where the desired temperature was achieved at a heating rate of 60 °C per hour.

### 2.3 Preparation of nano-porous samples

Opal films were prepared by a modified vertical deposition method based on isothermal heating evaporation-induced self-assembly.^21^ Obtained opal films were calcined at 850 °C for 12 hours, where the desired temperature was achieved at a heating rate of 60 °C per hour. Obtained pieces of opal film with mass of 100 mg were preliminarily dried in an oven at 300 °C for 3 hours and were then soaked in a 10 mL solution of crude oil (25 wt%) in trichloromethane. The obtained opal film impregnated with oil was dried under air flow for 2 hours. The oil content in a sample was measured by thermogravimetry (4 measurements) and appeared to be 6.9 ± 0.4%. This sample is denoted as “nano-porous” throughout the text.

### 2.4 Preparation of coarse-porous samples

The coarse-porous medium used as a reference sample in this study consisted of quartz sand sieved to a size of less than 40 μm with a BET surface area of 1.33 ± 0.07 m^2^ g^−1^. The porosity of the sample was determined by a water immersion technique according to the following eqn (1).1
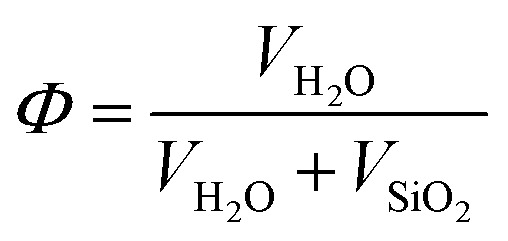
where *V*_H_2_O_ is the volume of water in an immersed sample (*ρ* = 0.997 g cm^−3^ at 25 °C), and *V*_SiO_2__ is the volume of silica in a sample (*ρ* = 2.65 g cm^−3^). The experiment was performed in triplicate and the average porosity value, with margins of error for a 95% confidence level, appeared to be 0.46 ± 0.03. The coarse-porous sample was prepared by mixing heavy oil with the pure quartz sand and is named “coarse-porous” throughout the text.

### 2.5 Thermal analysis

Simultaneous thermogravimetry-differential scanning calorimetry (TG-DSC) experiments were performed by using a STA 449 F1 Jupiter (Netzsch) thermoanalyzer over a temperature range of 30–600 °C. The experiments were conducted at linear heating rates of 5, 10, 15, and 20 K min^−1^, under air flow of 50 mL min^−1^ in alumina crucibles. In the case of the coarse-porous samples, the average mass of the samples was 10 mg. In the case of the nano-porous samples, obtained opal films containing oil were gently broken by a spatula into a small pieces (approx. 1 mm^2^ each) and samples with an average mass of approximately 10 mg were chosen for each run. Obtained DSC curves were processed using Proteus Analysis v5.2.1, NETZSCH Peak Separation (version 2010.09), and NETZSCH Thermokinetics 3.1 (version 06.08.2014) program packs.

### 2.6 Scanning electron microscopy

Scanning electron microscopy (SEM) measurements were carried out by using a field-emission high-resolution scanning electron microscope from Merlin Carl Zeiss. Observational photos of surface morphology were obtained at an accelerating voltage of incident electrons of 15 kV and a current probe of 300 pA. The average size of the silica spheres was measured from 100 measurements of individual spheres.

### 2.7 Nitrogen adsorption and desorption measurements

Nitrogen adsorption and desorption measurements at 77 K were carried out with an ASAP 2020 MP instrument (Micromeritics). Before the measurements, 0.5 g of sample was degassed by heating at 300 °C under vacuum (8 μm Hg) for 2 hours. The specific surface areas of the opal samples were determined by applying the Brunauer–Emmett–Teller (BET) equation. Pore size distribution was calculated from desorption curves by the Barrett–Joyner–Halenda (BJH) method with a cylindrical pore model.

## 3. Results and discussion

### 3.1 Obtaining and characterizing model porous media

Physical simulation of spatially confined processes requires the application of appropriate porous media. Most of the aforementioned studies^1–9^ use meso- and macroporous silica (SBA-15, MCM-41, CPG) as model porous media. Porous networks of these materials have been profoundly studied in numerous works, *e.g.*ref. 22–24.

Unlike the aforementioned model porous media, pore networks of shale samples are extremely complex.^25^ This complexity is caused by the fractal nature of the pore network^26,27^ including the presence of micro- (<2 nm), meso- (2–50 nm) and macropores (>50 nm) distributed within both the organic and inorganic parts of the shale.^28–30^ However, despite their simplicity, model porous media have been successfully applied for studying the influence of the nanoconfinement effect on physical processes.

In our work we propose synthetic opals as convenient porous media for studying spatially confined processes. The choice of synthetic opals as a model porous medium is related to their unique properties, which are: the regularity of pore distribution, the possibility of variation of the pore size in a wide range,^31^ the independence of the specific free volume from the pore size,^32^ high diffusion permeability,^32^ chemical inertness and excellent mechanical and thermal stability.^33^

**Fig. 1:**
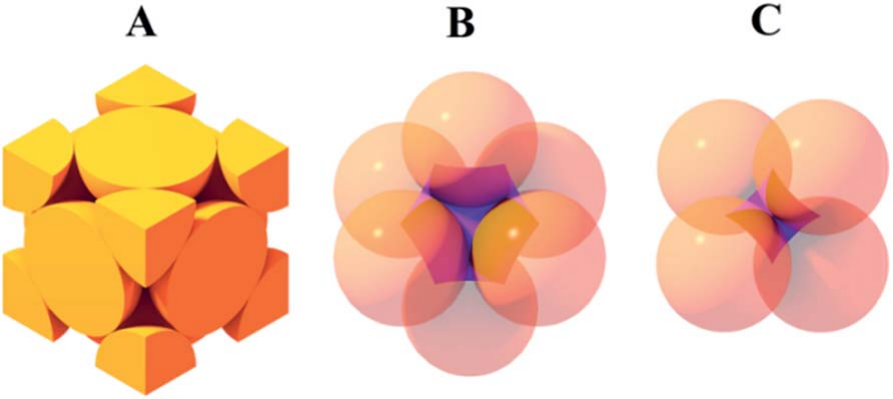
The unit cell of a face-centered cubic lattice (A). Tetrahedral (B) and octahedral (C) pores existing in the ideal opal crystal. The pores are colored purple.

Ideal opal crystals possess a face-centered cubic (FCC) lattice (Fig. 1A) containing two types of pores: tetrahedral and octahedral, formed by four (Fig. 1B) and six (Fig. 1C) touching spheres, respectively. These two types of pores are interconnected with each other and arranged into a pore network.

**Fig. 2:**
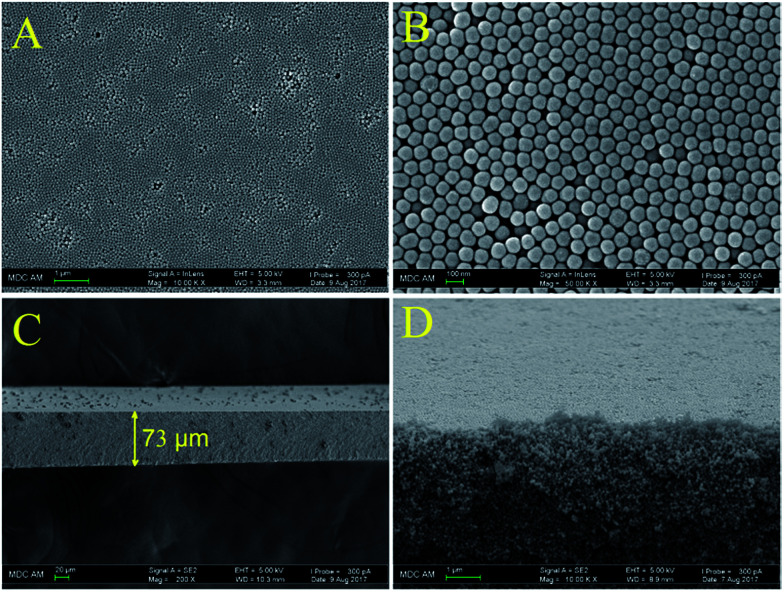
(A) and (B) {111} plane view of synthetic opal made of 90 nm silica spheres; (C): thickness of the obtained opal film; (D): close up of the edge of the opal film. Scale bars are 1 μm (A), 100 nm (B), 20 μm (C), and 1 μm (D).

Vertical deposition is a widely used method for production of colloidal crystals made of silica^34^ and polymeric nano- and microparticles.^35^ In this method strong capillary forces at a meniscus between a glass slide and a colloidal sol induce crystallization of spheres into a three dimensional close packed array. We used the modification of the vertical deposition method based on isothermal heating evaporation-induced self-assembly as a more reproducible and less time-consuming technique to allow us to produce well-ordered colloidal crystal films.^21^ We used SEM to examine the quality of the produced opals. The average size of the silica spheres was 93 ± 6 nm. Fig. 2 shows the morphology of the obtained opal films. The absence of cracks can be seen from the small scale images A and C. On the other hand large scale images B and D show the presence of a lot of point defects on a {111} plane of the crystal. The most probable reasons for such imperfectness are the polydispersity of the initial silica microparticles^21^ and the shrinkage of silica spheres during sintering.^33^ The obtained opal film appears to slightly increase in thickness from the top to the bottom in the direction of the solvent evaporation; the average thickness of the sample used in this research was approx. 70 μm.

**Fig. 3:**
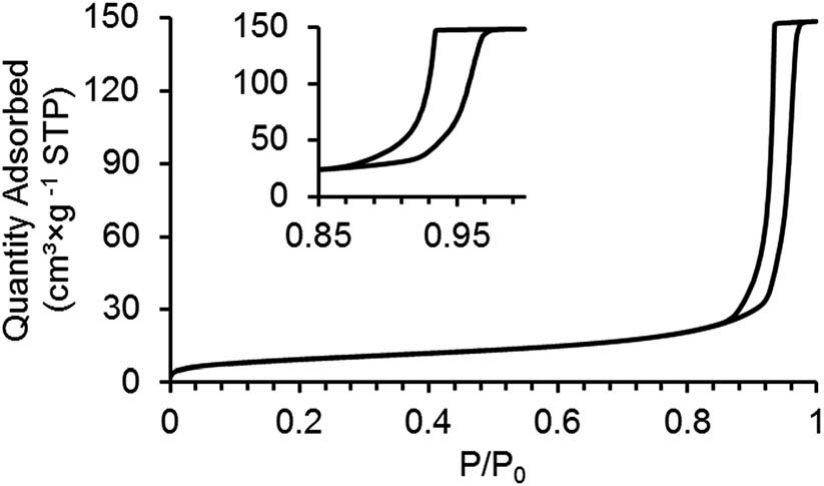
Nitrogen adsorption and desorption isotherms at 77 K and close up of the hysteresis loop (inset).

We used nitrogen adsorption–desorption measurements to study the textural features of the obtained synthetic opal. Nitrogen adsorption and desorption curves, as well as the hysteresis loop they form are presented in Fig. 3. It is clearly seen that the shape of the hysteresis loop corresponds to H2-type hysteresis (Fig. 3 inset) which is associated with porous materials containing mesoporous pore networks.^36^

**Fig. 4:**
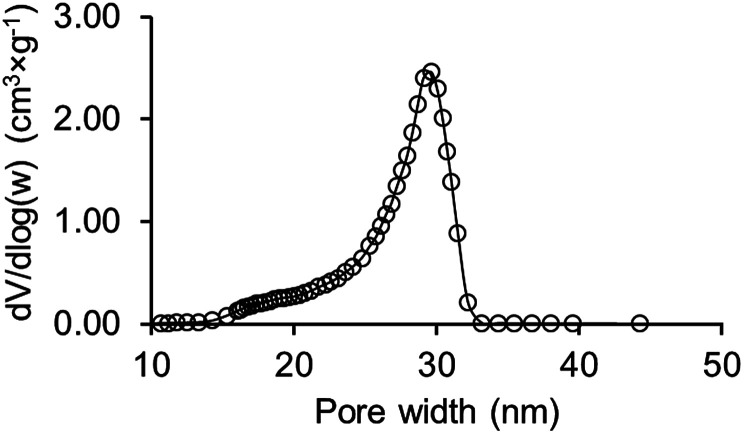
BJH pore size distribution curve of obtained synthetic opal.

The pore size distribution (PSD) was calculated from desorption data by the Barrett–Joyner–Halenda (BJH) method. As we can see from Fig. 4 PSD is presented by a narrow distribution curve with a maximum at 30 nm.

The value of the total pore volume of the synthesized sample is 0.23 ± 0.01 cm^3^ g^−1^ which corresponds to porosity of 0.37 (skeletal density of silica nanoparticles is 2.17 g cm^−3^).^33^ The obtained value is higher than the theoretical value of 0.26 for an ideal opal crystal; and this difference in porosity might be related to the aforementioned packing defects formed during opal crystal growth and its further sintering. The BET surface area for synthesized sample is 33.78 ± 0.20 m^2^ g^−1^. The coarse-porous medium possesses a much lower surface area of 1.33 ± 0.07 m^2^ × g^−1^ and higher porosity of 0.46 ± 0.03 (see Materials and methods section).

### 3.2 Combustion study by differential scanning calorimetry and kinetic analysis

The next step was the impregnation of oil into the synthetic opal’s pores. We used a heavy oil solution in chloroform for saturation of the sample. The oil content in the final sample was evaluated by TG measurements and was 6.9 ± 0.4%. Based on oil density (Table 1), and opal porosity, calculated from the data of nitrogen porosimetry, we estimated the degree of pore filling, which appears to be 33%. It should be noted that the oil impregnated into opals may slightly differ from that of the bulk sample due to adsorption of polar compounds (resins and asphaltenes) onto the silica surface.

**Fig. 5:**
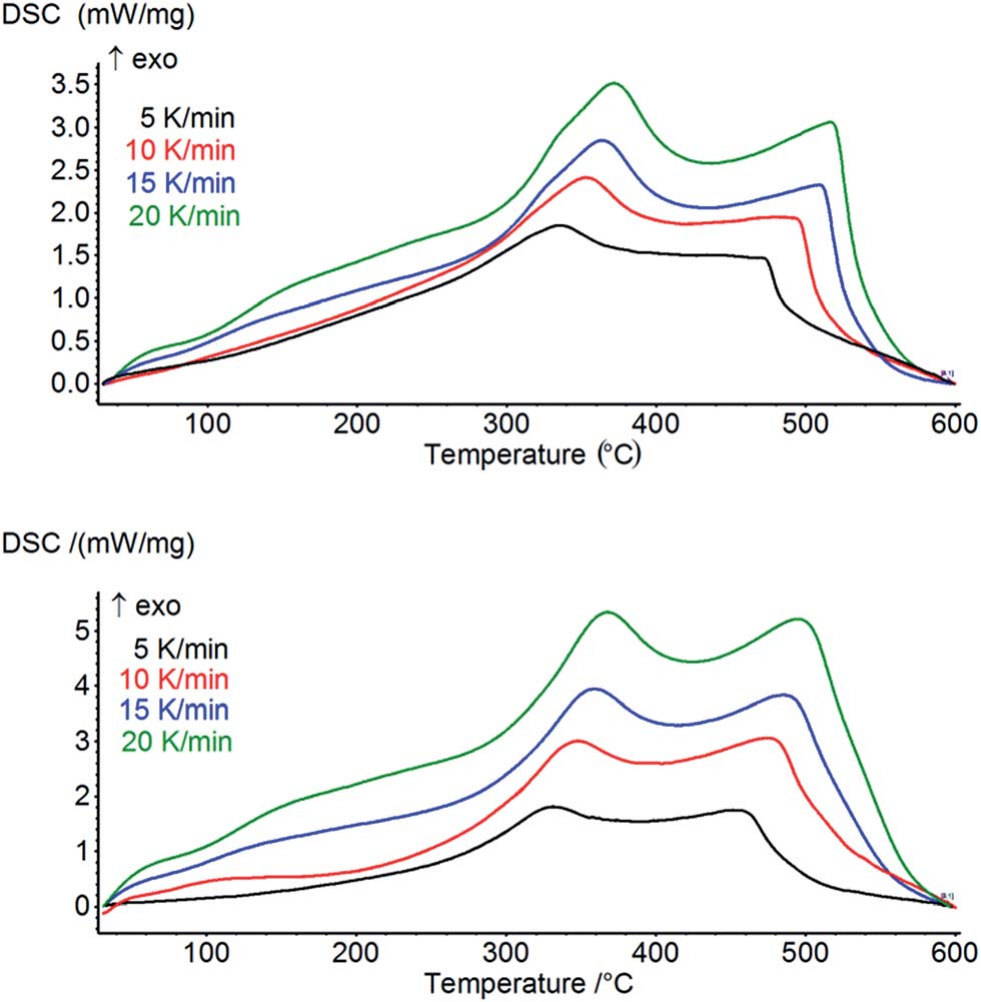
DSC curves for heavy oil oxidation in coarse-porous (top) and nano-porous media (bottom).

We used differential scanning calorimetry (DSC) to study differences in the kinetics of heavy oil oxidation in coarse-porous and nano-porous media. As a reference, we used a coarse-porous sample mixture made of pure quartz sand and the same heavy oil (oil content is 7.1 ± 0.3%). DSC curves related to heavy oil combustion for both samples performed at different heating rates are given in Fig. 5. The figure shows the presence of two main oxidation regions, namely low temperature oxidation (LTO) and high temperature oxidation (HTO). LTO yields oxygenated hydrocarbons (peroxides, alcohols and carbonyl compounds) without producing significant amounts of carbon oxides, and HTO is usually described as coke oxidation, producing carbon oxides and water.^37^

**Fig. 6:**
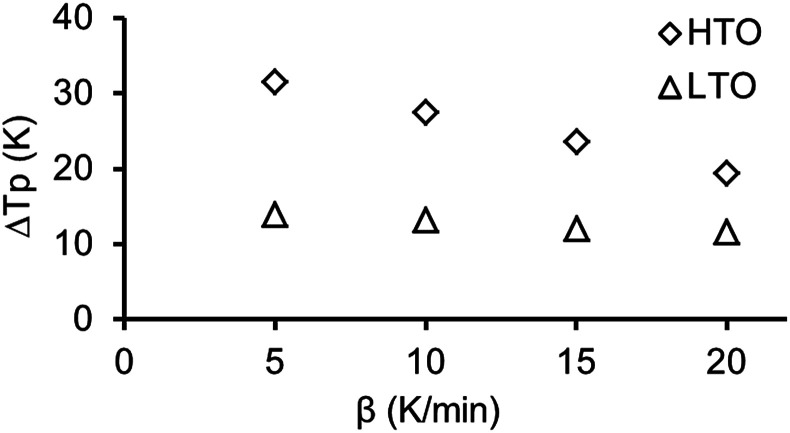
Differences between the peak temperatures (Δ*T*_p_) of heavy oil combustion in coarse-porous and nano-porous media for low and high temperature oxidation at different heating rates.

Fig. 6 shows the differences between corresponding pairs of *T*_p_ values for low and high temperature oxidation. From Fig. 6 one can clearly see the peak temperatures of nanoconfined oil combustion are significantly lower for both LTO and HTO compared with those of the coarse-porous sample which evidences that the reaction in nanoporous medium proceeds faster. The differences between *T*_p_ become smaller with the increase of the heating rate from 5 to 20 K min^−1^ for both low and high temperature oxidation processes.

Oil oxidation can be described as a simple reaction rate model presented by eqn (2), that assumes functional dependency on oil conversion degree *α*, and oxygen partial pressure:^37^2
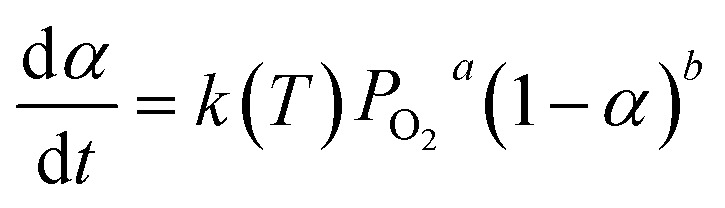


The rate constant *k*(*T*) is assumed to obey the Arrhenius law (eqn (3)):3
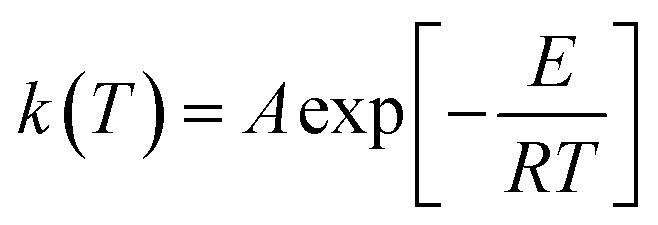


The values of *α* are determined as fractional areas of the DSC peak.

Early studies^38,39^ show a first-order dependency of oil combustion rate with respect to both oil concentration and oxygen partial pressure. Since small amounts of oil were used for each DSC run in our study (about 0.7 mg) and a high air flow rate of 50 mL min^−1^ was combined with a large furnace volume (approx. 250 mL), we assumed that oxygen partial pressure remained constant (21.2 kPa) during the experiment. Considering all of the above, we express the final reaction model in eqn (4).4
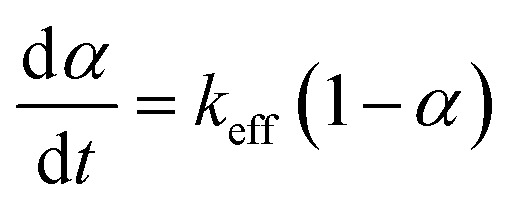
where *k*_eff_ = *kP*_O_2__.

We used Kissinger’s method^40^ for calculation of the kinetic parameters of the combustion processes. The basic equation of the method is derived from eqn (5) under the conditions of the maximum reaction rate (d*α*/d*t* = max, therefore d^2^*α*/d*t*^2^ = 0), eqn (6).5
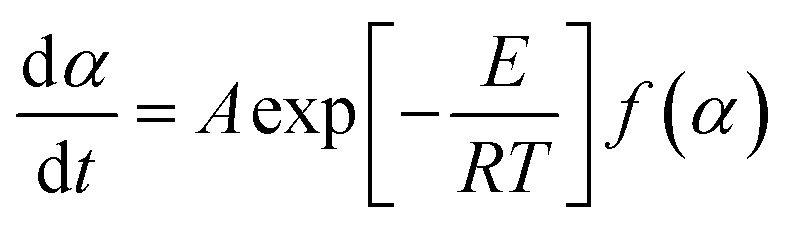
6
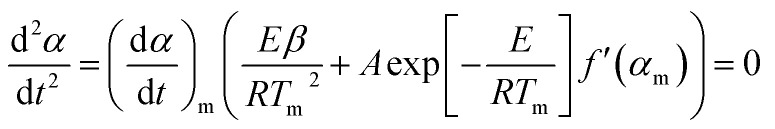
where *f*′(*α*) = d*f*(*α*)/d*α*, *β* = d*T*/d*t*, and the index “m” denotes the values related to the maximum rate. It follows from eqn (6) that:7
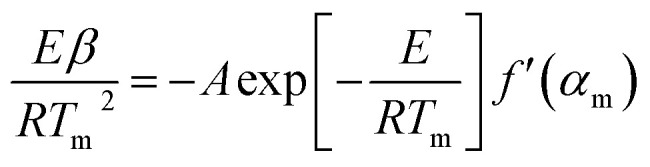
or in the logarithmic form:8
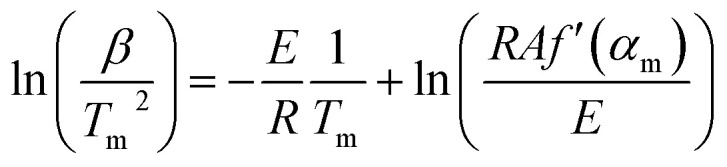


The main limitation of the method is that the determination of an activation energy value requires *f*′(*α*) to be independent of the heating rate. Otherwise, the second term on the right hand side of eqn (8) would not be constant and the plot of ln(*β*/*T*_m_) *vs.* 1/*T*_m_ would systematically deviate from a straight line, producing a systematic error in activation energy.^41^ Strict independence of *f*′(*α*_m_) from *β* is accomplished for a first order kinetic model *f*(*α*) = 1 − *α* (*f*′(*α*) = −1), which is consistent with the chosen kinetic model (eqn (4)). The final form of the Kissinger method is presented by eqn (9).9
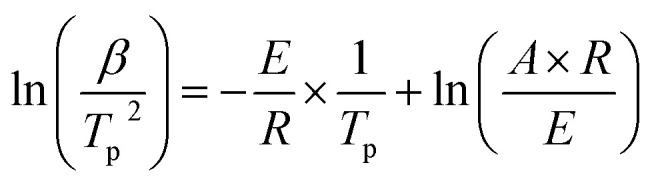


We decided to use Kissinger’s method for calculation of kinetic parameters of thermally stimulated processes over other integral^42,43^ and differential methods^44^ due to the significant overlapping of DSC signals of the evaporation and the low and high temperature oxidation processes. Such overlapping makes the results of kinetic calculations dependent on parameters of the peak separation procedure, like the peak profile or the type of baseline. The Kissinger method uses peak temperature values (*T*_p_) for calculation of kinetic parameters that are almost independent of the baseline choice and peak profile.

**Fig. 7:**
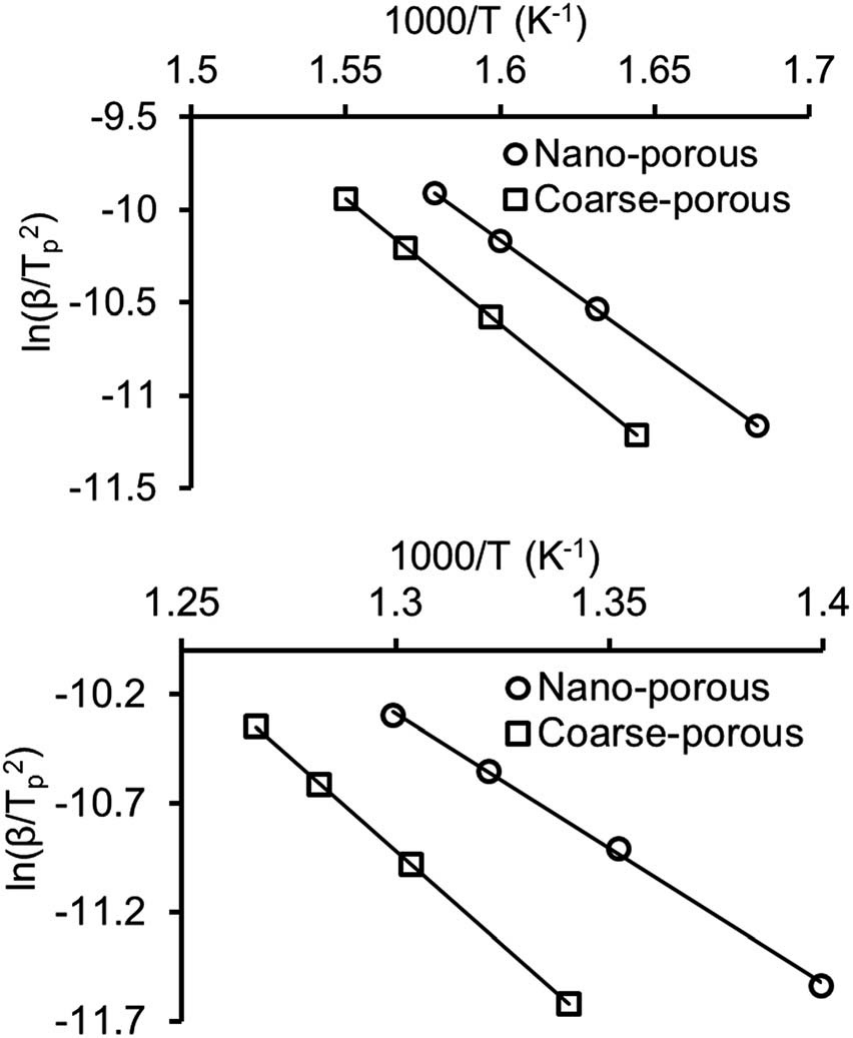
Kissinger plots for heavy oil oxidation in nano-porous and coarse-porous media (top is for LTO, bottom is for HTO).

Fig. 7 shows Kissinger plots for low and high temperature oxidation in both porous media. The obtained plots demonstrate statistically significant linearity with correlation coefficients *R*^2^ not less than 0.995. The calculated kinetic parameters, with margins of error for a 95% confidence level, are grouped in Table 2. From Table 2 it can be seen that nanoconfinement lowers the effective activation energy for both LTO and HTO.

**Table 2:** The kinetic parameters of the combustion processes

	Coarse-porous		Nano-porous	
	LTO	HTO	LTO	HTO
*E*_a_, kJ mol^−1^	111.8 ± 0.3	144.6 ± 3.4	100.4 ± 3.2	103.0 ± 6.1
log *A*, [*A*] in min^−1^	8.9 ± 0.1	9.3 ± 0.2	8.0 ± 0.2	6.6 ± 0.4

It should be noted that the calculated effective *A* and *E*_a_ parameters make opposite contributions to the reaction rate constant *k*(*T*) for both LTO and HTO processes. In the case of LTO, the nanoconfined process proceeds with higher *E*_a_ (which decreases *k*(*T*)) and higher pre-exponential factor *A* (which increases *k*(*T*)) compared with the reaction in the coarse-porous medium. A similar situation occurred for HTO: the nanoconfined reaction possesses lower *E*_a_ (increasing *k*(*T*)) and lower *A* (decreasing *k*(*T*)) compared with the process in the coarse-porous medium.

**Fig. 8:**
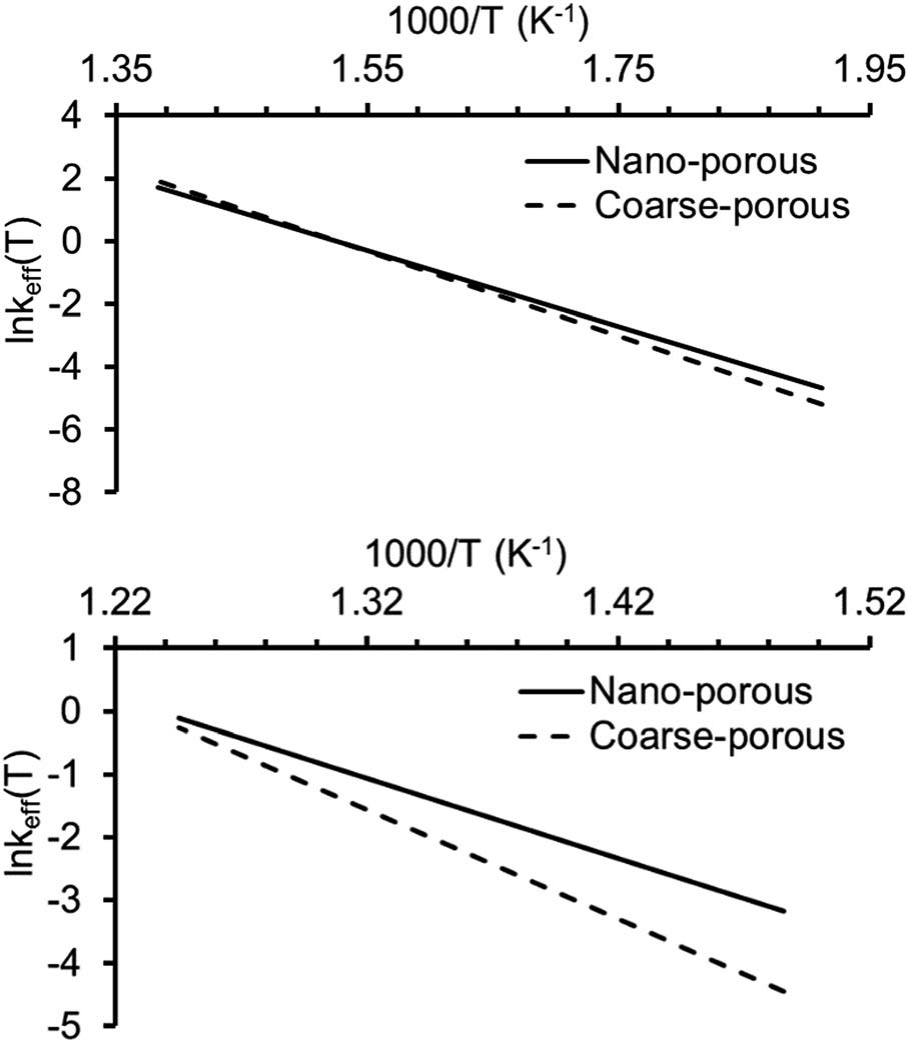
Variation of effective rate constants with temperature for heavy oil oxidation in nano-porous and coarse-porous media (top is for LTO, bottom is for HTO).

To estimate the combined effect of these two parameters we calculated *k*(*T*) values for LTO and HTO over the temperature ranges where these processes occurred. Comparison of effective rate constants for heavy oil oxidation in nano-porous and coarse-porous media are presented in Fig. 8. One can see the rates of low temperature oxidation in both porous media are practically the same, whereas the HTO process proceeds faster in the nanoporous medium than in the coarse-porous medium, especially at low temperatures. The results are consistent with the DSC data that show that nanoconfinement predominantly influences HTO.

Our current study shows that the textural parameters of porous media influence the reactivity of crude oil in combustion process. Since the simulation of *in situ* combustion processes require reliable kinetic parameters, for the proceeding chemical reactions, to allow for trustworthy prediction, this influence should be taken into consideration. For solid state processes the influence of nanoconfinement can be divided into surface and size effects.^45^ In the case of the surface effect, changes in the behavior of the confined substrate are caused by strong interaction of a substrate with the surface. In the case of the size effect, these changes are related to an increase of the contribution of the surface free energy relative to the free energy of the bulk phase due to a decrease in the size of the nanocrystals. Although the investigation of mechanisms of the influence of porous media on combustion process lies far beyond the scope of this study, we suggest some possible reasons for the combustion acceleration in the nano-porous medium that we observed in the case of high temperature oxidation. Since HTO is usually described as a heterogeneous coke oxidation process,^37^ we suggest that the well developed surface of the synthetic opal can affect the reactivity of coke produced during the pyrolysis stage. Another reason is that the produced coke is spread over a larger silica surface area, compared with the coarse-porous medium, increasing the interface area between the coke and the air, which favors oxidation. These suggestions resonate with previously published studies on the influence of surface area of different minerals on heavy oil pyrolysis and combustion processes. It was shown that the addition of minerals with highly developed surfaces increase the amount of coke formed during the pyrolysis stage,^46,47^ and a decrease in the oil/surface ratio results in a reduction of the effective activation energy of the combustion process.^48^

## 4. Conclusions

We prepared and used synthetic opals as convenient model porous media to study the confinement effect on the chemical reactivity of oil in the combustion process. We showed that a modified vertical deposition technique can be easily applied for production of synthetic opals with the desired pore size and a narrow pore size distribution. The kinetics of heavy oil combustion confined in 30 nm pores was studied by DSC with application of non-isothermal kinetic analysis. The comparison of kinetic parameters of the oil oxidation process for nano-porous and coarse-porous media revealed that nanoconfinement predominantly influences the high temperature oxidation process.

## Conflicts of interest

There are no conflicts to declare.

## Supplementary Material
